# Dysregulation of Blimp1 transcriptional repressor unleashes p130Cas/ErbB2 breast cancer invasion

**DOI:** 10.1038/s41598-017-01332-z

**Published:** 2017-04-25

**Authors:** Marianna Sciortino, Maria del Pilar Camacho-Leal, Francesca Orso, Elena Grassi, Andrea Costamagna, Paolo Provero, Wayne Tam, Emilia Turco, Paola Defilippi, Daniela Taverna, Sara Cabodi

**Affiliations:** 1 Department of Biotechnology and Health Sciences, University of Turin, Via Nizza 52, 10126 Turin, Italy; 2 Department of Pathology and Laboratory Medicine, Weill Cornell Medicine, New York, NY USA

## Abstract

ErbB2 overexpression is detected in approximately 20% of breast cancers and is correlated with poor survival. It was previously shown that the adaptor protein p130Cas/BCAR1 is a crucial mediator of ErbB2 transformation and that its overexpression confers invasive properties to ErbB2-positive human mammary epithelial cells. We herein prove, for the first time, that the transcriptional repressor Blimp1 is a novel mediator of p130Cas/ErbB2-mediated invasiveness. Indeed, high Blimp1 expression levels are detected in invasive p130Cas/ErbB2 cells and correlate with metastatic status in human breast cancer patients. The present study, by using 2D and 3D breast cancer models, shows that the increased Blimp1 expression depends on both MAPK activation and miR-23b downmodulation. Moreover, we demonstrate that Blimp1 triggers cell invasion and metastasis formation via its effects on focal adhesion and survival signaling. These findings unravel the previously unidentified role that transcriptional repressor Blimp1 plays in the control of breast cancer invasiveness.

## Introduction

The amplification or overexpression of the tyrosine kinase receptor ErbB2 accounts for approximately 20% of all breast cancers^1^, and ErbB2 amplification is detected in about 50% of ductal carcinomas *in situ* (DCIS) of the mammary gland^2^. This implies that the aggressive invasive phenotype that is associated with ErbB2 is not solely due to its overexpression, but that additional factors are required before the transition towards invasive carcinoma occurs^3^. However, the mechanisms that underlie the progression towards invasive tumor formation are still unclear.

We have already demonstrated that the adaptor protein p130Cas/BCAR1 (Crk associated substrate/Breast Cancer Anti-estrogen Resistance protein 1) plays a key role in the control of migration and invasion in ErbB2 positive breast cancer^4, 5^. It is also well known that 3D cultures of MCF10A.B2 mammary epithelial cells, which contain a chimeric and activatable ErbB2 receptor, can form spheroid structures called acini^6^, meaning that these structures recapitulate the architecture of the ductal lobular unit in the human mammary gland and can therefore be considered a faithful model with which to study mammary gland biology *in vitro*. We have previously shown that MCF10A.B2 cells are able to develop invasive protrusions upon the concomitant activation of ErbB2 and overexpression of the p130Cas protein^5^. Moreover, *in vivo* analyses of human breast cancer have confirmed that the amplification of ErbB2 in combination with the overexpression of p130Cas induces a higher proliferation rate and an increased number of distant metastases as well as correlating with poor prognosis^5, 7^. At the molecular level, the invasive behavior resulting from the p130Cas/ErbB2 interaction relies on the activation of AKT/PI3K and Erk1/2 MAPKs signaling pathways^8^.

An analysis of the transcriptional changes that occur during p130Cas/ErbB2 invasion in MCF10A.B2 spheroids^4^, has highlighted the upregulation of PRDM1 (PR domain containing 1) mRNA in p130Cas/ErbB2 invasive acini. The PRDM1 gene encodes for the human Blimp1 protein (B-lymphocyte-induced maturation protein-1) and its role as a transcriptional repressor in the immune system has been widely studied^9^. Indeed, Blimp1 is able to recruit chromatin modifiers, such as methyltransferases and deacetylases, that can, in turn, regulate B and T cell differentiation^10^. The function of Blimp1 in cell migration during the developmental and physiological processes has been described in a number of animal models and tissues^11–13^. In pathological conditions, Blimp1 acts as a tumor suppressor in different types of lymphomas from B, T and NK cells^14, 15^, however, only a small number of reports have discussed its function in non-hematopoietic tumors^16–18^. In fact, Blimp1 has been reported to mediate EMT upon TGF-β1 stimulation in ER-negative breast cancer cells by repressing BMP-5 and, in turn, activating the transcription factor Snail^16^. Moreover, Blimp1 has been found to act as a mediator of Ras/Raf/AP-1 signaling in lung cancer cell lines^18^.

Importantly, the non-coding signature that characterizes p130Cas/ErbB2 invasive behavior has highlighted miR-23b role as a putative regulator of Blimp1 expression. Indeed, miR-23b has been found to be downmodulated in invasive p130Cas/ErbB2 acini and bioinformatics analyses have proposed Blimp1 as a putative miR-23b target gene^4^. miR-23b belongs to the miR-23b~27b~24-1 cluster which is intronically present on chromosome 9 of the aminopeptidase O gene^19^. The function of miR-23b in breast cancer pathogenesis is still debated, as some reports attribute miR-23b with a tumor suppressor role, while others suggest that its oncogenic function depends on cellular model^20^.

Our results provide evidence of a new-found, pro-invasive function for Blimp1 in p130Cas/ErbB2 invasive breast cancer and describes its regulation, mechanism of action and *in vivo* functions. Moreover, it is demonstrated, for the first time in a non-hematopoietic system, that miR-23b is a direct Blimp1 regulator, a discovery that provides insights into miR-23b target genes in breast cancer.

## Results

### p130Cas and ErbB2 induce Blimp1 overexpression in human invasive breast cancer via Erk1/2 MAPKs pathway activation

We have previously shown that MCF10A.B2 human mammary acini grown in 3D form polarised, quiescent single acini, that upon activation of ErbB2 with the synthetic ligand AP1510, undergo proliferation and disruption of apical polarity, forming multiacinar structures. The overexpression of p130Cas in MCF10A.B2 cells is sufficient to give rise to multiacinar structures that upon stimulation with AP1510 acquire invasive protrusions^8^. The gene signature underlying the transition from multiacinar structures, characterized by p130Cas overexpression or ErbB2 activation, to invasive p130Cas/ErbB2 acini has also been identified^4^. The transcriptional repressor Blimp1/PRDM1 mRNA was found to be upregulated in invasive acini. We therefore decided to explore its involvement in p130Cas/ErbB2 mediated breast cancer invasion.

**Figure 1:**
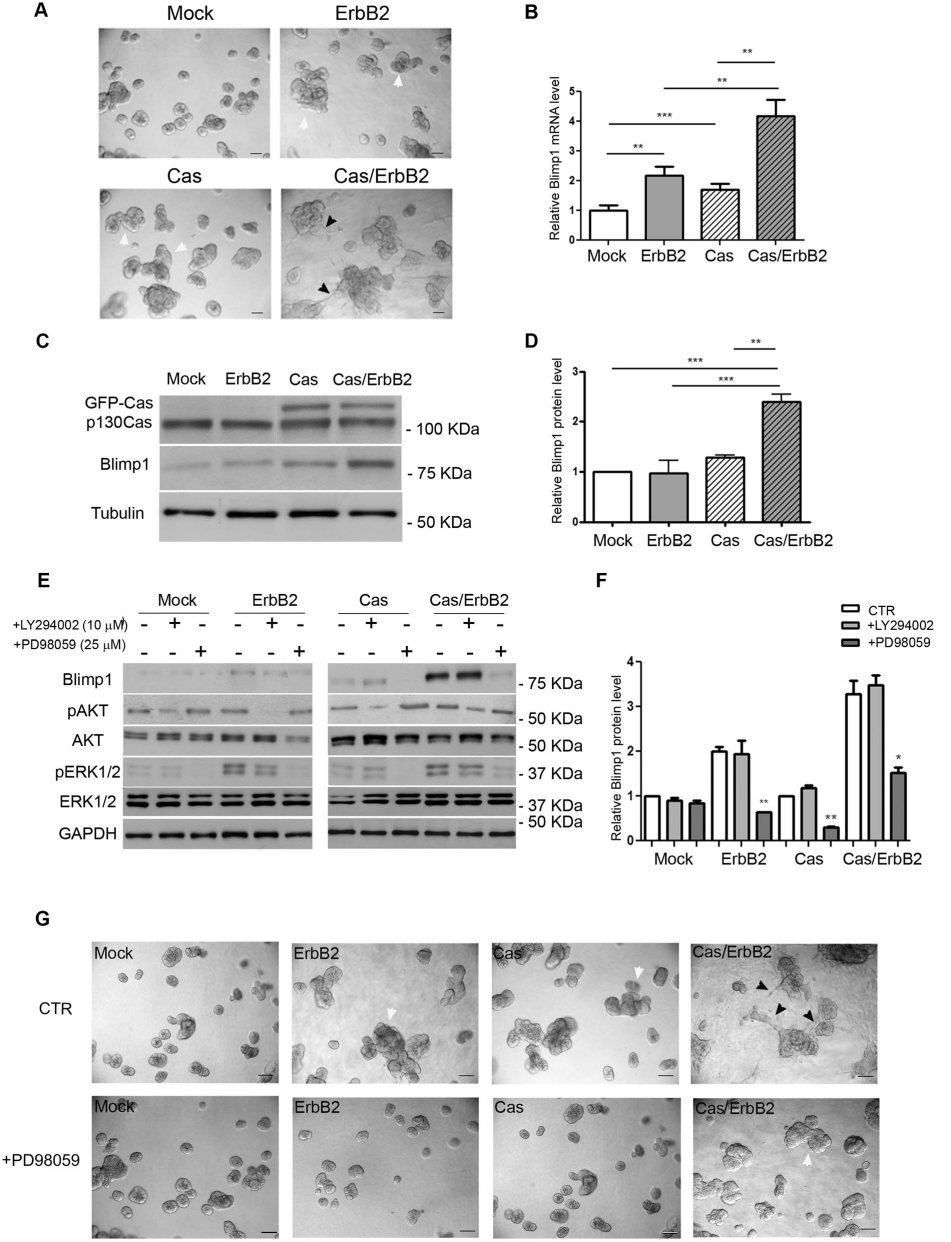
High expression of Blimp1 protein in p130Cas/ErbB2 invasive acini is sustained by Erk1/2 activation. (**A**) Day 14 culture of control (Mock) and p130Cas overexpressing MCF10A.B2 (Cas) plated on a matrigel:collagen matrix and treated with either 1µM ethanol (vehicle) or AP1510 on day 10 to activate ErbB2 (ErbB2; Cas/ErbB2). Scale bars, 50µm. Magnification 20X. White arrowheads indicate multiacinar structures. Black arrowheads indicate invasive acini. (**B**) On day 14 acinar structures were recovered and lysed and Blimp1 mRNA expression levels were evaluated by RT-PCR. Statistical analysis was performed using the Student's t-test (*p < 0.05, **p < 0.01, ***p < 0.001). (**C**) Day 14 protein extracts were probed for Blimp1 in a western blot analysis. Tubulin was used as the loading control. (**D**) Densitometric analysis of protein levels of at least three independent experiments is shown (mean ± s.e.m). Blimp1 protein modulation was calculated relative to Mock level and normalized on tubulin as the loading control. Statistical analyses were performed using the Student’s t-test (**p < 0.01, ***p < 0.001). (**E**) Acini derived from Mock and Cas cells were treated on day 10 with either DMSO (vehicle), 10 µM LY294002 (PI3K inhibitor) or 25 µM PD98059 (MAPK pathway inhibitor) in both in the presence and absence of 1 µM of AP1510. After 4 days of treatment, acini were recovered and lysed. Total cell extracts were probed for Blimp1 in a western blot analysis. GAPDH was used as loading control. (**F**) Densitometric analysis of protein levels of at least three independent experiments is shown (mean ± s.e.m). Protein modulation was calculated relative to Mock level and normalized to GAPDH as the loading control. Statistical analyses were performed using the Student’s t-test (*p < 0.05, **p < 0.01). (**G**) Representative phase images of day 14-acinar structures showing invasive protrusion impairment upon AKT/PI3K and MAPK pathway inhibition. Scale bars, 50 µm. Magnification 20X. White arrowheads indicate multiacinar structures. Black arrowheads indicate invasive acini.

Blimp1 mRNA and protein expression levels were thus evaluated in control MCF10.B2 cells grown in 3D (Mock, Fig. 1A), in ErbB2 that was activated by treatment with the AP1510 homodimerizer (ErbB2, Fig. 1A), in Cas overexpressing (Cas; Fig. 1A) and in invasive Cas overexpressing and ErbB2 activated acini (Cas/ErbB2; Fig. 1A). As shown in Fig. 1B, the mRNA levels of PRDM1, as measured by qRT-PCR, are significantly upregulated, relative to controls, in multiacinar structures that result from ErbB2 activation or p130Cas overexpression. Moreover, even higher PRDM1 mRNA level induction was observed in Cas/ErbB2 invasive acini, indicating the existence of a synergistic effect between ErbB2 activation and p130Cas overexpression. In fact, Blimp1 protein levels were found to be significantly enhanced in p130Cas/ErbB2 invasive acini (Fig. 1C and D). Interestingly, we did not observe a perfect match between RNA and protein levels in p130Cas overexpressing and ErbB2 activated multiacini, suggesting the existence of additional post-transcriptional regulation mechanisms.

Our previous work has demonstrated that high levels of p130Cas expression, in the presence of ErbB2 activation, in 3D acini trigger the activation of both the PI3K/Akt and MAPK signaling pathways^8^. It was therefore decided to investigate the possible involvement of these two pathways in Blimp1 expression modulation. To this end, Mock and Cas acini, stimulated or not with AP1510 to trigger ErbB2 activation, shown in Supplementary Figure 1, were treated with either LY294002 (10 µM) or PD98059 (25 µM) to inhibit the PI3K and MAPK pathways, respectively. As shown in Fig. 1E,F and in Supplementary Figure 2, Blimp1 expression in Cas/ErbB2 multiacinar and invasive structures is preferentially modulated upon MAPK pathway activation. Indeed, while treatment with the PI3K inhibitor LY294002 gave no significant changes in Blimp1 expression levels, the addition of MAPK inhibitor PD98059 led to a reduction in these levels in all conditions, thus suggesting that MAPKs are a major regulator of Blimp1 expression. Interestingly, the reduction in Blimp1 expression upon MAPK inhibitor treatment resulted in a reduced formation of ErbB2 and p130Cas multiacinar structures and in the abrogation of invasive protrusions in Cas/ErbB2 acini (Fig. 1G and Supplementary Figure 3 for quantification). These data indicate that the activation of MAPK signaling induces Blimp1 expression in both multiacinar and invasive structures. Consequently, the pharmacological inhibition of MAPK signaling results in the impairment of multiacinar and invasive structure formation.

### Modulation of Blimp1 expression affects p130Cas/ErbB2 dependent invasion *in vitro*

**Figure 2:**
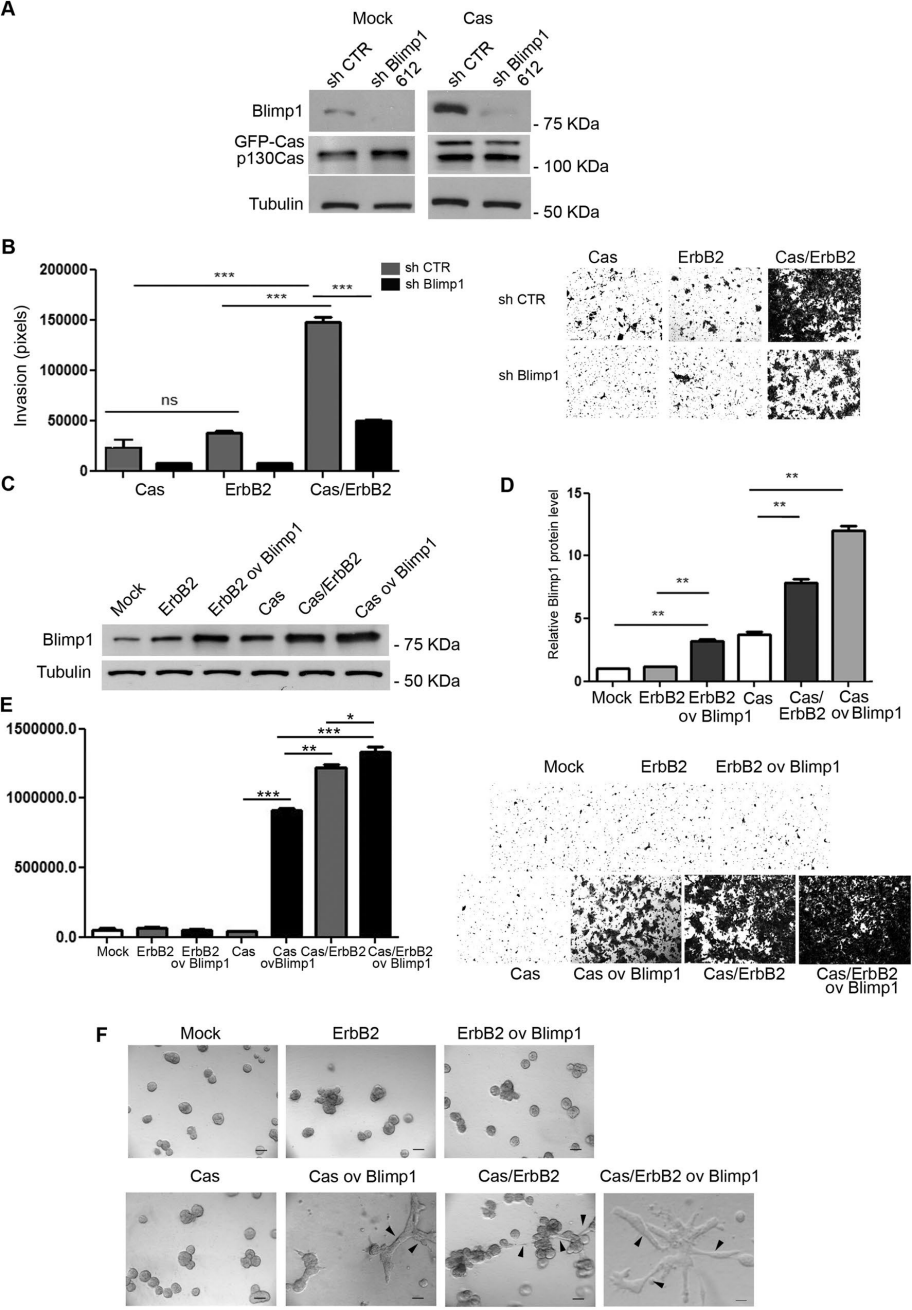
Blimp1 expression modulation influences migratory capacity in 2D and 3D cultured cells. (**A**) Mock and Cas cells were transduced with the sh Blimp1 clone 612 vector and the Blimp1 and p130Cas protein levels were evaluated using western blot analysis. Tubulin was used as the loading control. (**B**) Mock and Cas cells, that had either been transduced with human sh Blimp1 clone 612 vector or scrambled ctr vector, were subjected to transwell invasion assays for 48 hours both in the presence and absence of 1 µM AP1510. Cell invasion quantification was performed over three different experiments. Results are presented as mean ± s.e.m. of the area covered by invaded cells. Representative images of invaded cells after 48 hours, fixed and stained with Crystal violet are shown (***p < 0.001). Magnification 5X. (**C**) Mock and Cas cells were transduced with Blimp1 overexpressing vector and treated or not with 1 µM AP1510 for 48 hours. Total cell extracts were probed for Blimp1 in a western blot analysis. Tubulin was used as the loading control. (**D**) Densitometric analyses of protein levels of at least three independent experiments are shown (mean ± s.e.m). Blimp1 protein modulation was calculated relative to Mock level and normalized to tubulin as the loading control. Statistical analyses were performed using the Student’s t-test (**p < 0.01). (**E**) Invasion of Blimp1 overexpressing ErbB2, Cas and Cas/ErbB2 cells was evaluated by transwell invasion assays in the presence or absence of 1 µM AP1510. Results are presented as mean ± s.e.m. of the area covered by invaded cells. Representative images of invaded cells after 48 hours, fixed and stained with Crystal violet are shown (*p < 0.05, **p < 0.01,***p < 0.001). Magnification 5X. (**F**) Representative phase contrast images of acinar structures taken at day 14 are presented. Magnification 20X, scale bars 50 µm. Black arrowheads indicate invasive acini.

In order to investigate whether Blimp1 is required for p130Cas/ErbB2 mediated invasion, Mock and Cas overexpressing MCF10.B2 cells were infected with lentiviruses that expressed either Blimp1 or scrambled control shRNA sequences (Fig. 2A) and assessed for their invasive behavior by performing Transwell invasion assays in transwell-coated with matrigel/collagen (Fig. 2B). These experiments indicate that the lowering of Blimp1 levels is sufficient to impair the invasive phenotype driven by p130Cas overexpression and ErbB2 activation (Fig. 2B).

To exclude the off-target effects of the shBlimp1 sequence, another sh sequence was tested and it was confirmed that lowering Blimp1 expression does causes severe alterations in cell invasion and does not alter p130Cas expression (Supplementary Figure 4A,B). To test whether Blimp1 overexpression alone is able to trigger invasion in non-invasive Cas and ErbB2 multiacinar structures, we generated lentiviruses that overexpressed Blimp1 and infected Mock and Cas cells, that were either subsequently treated with the ErbB2 homodimerizer or left untreated (Fig. 2C and D). Transwell invasion assays and 3D cell cultures revealed that Blimp1 overexpression is sufficient to induce invasion in Cas overexpressing cells that usually give rise to non-invasive multiacinar structures (see Fig. 1A). Conversely, Blimp1 overexpression is not able to induce either multiacinar structures or invasion in Mock (Supplementary Figure 5) and in ErbB2 transformed cells in itself, suggesting that p130Cas overexpression is required for cell invasion (Fig. 2E,F). Notably, the activation of ErbB2 in Cas cells that overexpress Blimp1 further increases their invasive capability, indicating a synergistic effect of p130Cas and ErbB2 in Blimp1-dependent cell invasion. These data indicate that high levels of Blimp1 in p130Cas overexpressing cells trigger the transition of breast epithelial cells from a non-invasive to a migratory and invasive cell phenotype and that the activation of ErbB2 in this context amplifies the invasiveness.

### Silencing Blimp1 expression impairs focal adhesion formation

p130Cas has been described as playing an active role in cell migration and invasion by regulating FAK (Focal Adhesion Kinase) and focal adhesion dynamics^21–24^, and it has also been shown that the recruitment of p130Cas and FAK at focal adhesions is required for proper extracellular matrix degradation and, consequently, efficient cancer cell invasion^25–28^. It was therefore decided to investigate whether FAK is implicated in the mechanism that underlies Blimp1-dependent cell invasion in p130Cas overexpressing and ErbB2 transformed mammary epithelial cells.

**Figure 3:**
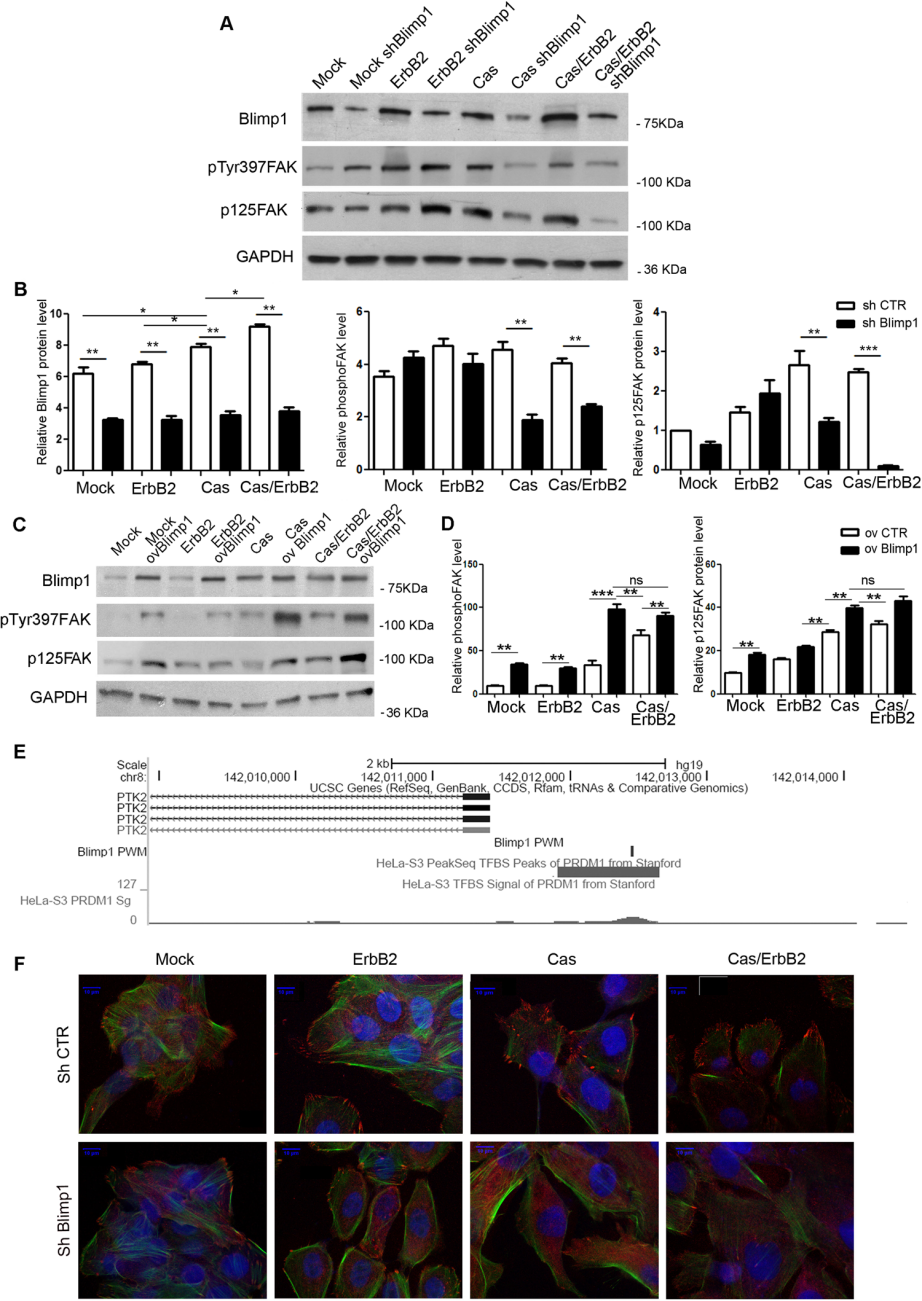
Focal adhesion protein expression is altered upon Blimp1 silencing. (**A**) Mock and Cas cells were transduced with the sh Blimp1 vector and treated with 1 µM AP1510 for 48 hours. Total cell extracts were probed for Blimp1, phospho-p125Fak(Tyr397), p125FAK, in a western blot analysis. GAPDH was used as the loading control. (**B**) Densitometric analysis of protein levels in at least three independent experiments is shown (mean ± s.e.m). Protein modulation was calculated relative to Mock level and normalized to GAPDH as the loading control. Statistical analyses were performed using the Student’s t-test (*p < 0.05, **p < 0.01,***p < 0.001). (**C**) Mock and Cas cells were transduced with Blimp1 overexpressing vectors and treated or not with 1 µM AP1510 for 48 hours. Total cell extracts were probed for Blimp1, phospho-p125Fak(Tyr397) and p125FAK in a western blot analysis. GAPDH was used as the loading control. (**D**) Densitometric analyses of protein levels in at least three independent experiments are shown (mean ± s.e.m). Phospho-p125Fak(Tyr397) and p125FAK modulation was calculated relative to Mock level and normalized to GAPDH as the loading control. Statistical analyses were performed using the Student’s t-test (**p < 0.01). (**E**) The core promoter of PTK2 (FAK) harbors a Blimp1 PWM match (rectangle) with a log-likelihood of 13.79, in correspondence with a ChiP-seq peak identified in the HeLa cells. (**F**) Immunofluorescence staining for vinculin (red) and phalloidin (green) of Mock and Cas MCF10A.B2 treated or not with 1 µM AP1510 for 48 hours. Scale bars: 10 µm. Magnification 63X.

As shown in Fig. 3A and B, FAK expression levels and activation were significantly reduced in Cas overexpressing cells silenced for Blimp1 but not in ErbB2 cells. Noteworthy, the downregulation of FAK expression and activity levels was even stronger in Cas/ErbB2 cells, thereby further indicating that p130Cas per se is sufficient to exert a regulatory mechanism on FAK by Blimp1 while ErbB2 can contribute to FAK regulation by Blimp1 only in presence of p130Cas. Consistently, Blimp1 overexpression in Cas cells strongly enhanced FAK activation and expression levels (Fig. 3C,D and Supplementary Figure 6). The fact that Blimp1 overexpression significantly induces FAK expression and activation also in Mock and ErbB2 cells supports a more general role for Blimp1 in FAK expression regulation (Fig. 3C,D). Interestingly, *in silico* analysis searching for Blimp1 binding sites on FAK promoter identified 1133 base pairs upstream of the FAK transcription start site, a ChIP-seq peak for PRDM1 by the Encode project (Fig. 3E), this peak also contains a DNA sequence that has a high log-likelihood (13.79) for the PRDM1 PWM, further supporting the experimental evidence of PRDM1 binding (Fig. 3E)^29, 30^. Notably, Blimp1 silenced Cas/ErbB2 cells that do not show invasive properties display impaired focal adhesion distribution and FAK localization compared to invasive Cas/ErbB2 cells (Fig. 3F and Supplementary Figure 7). Consistently, the overexpression of Blimp1 in Cas/ErbB2 cells further enhances focal adhesion structures (Supplementary Figure 8). These data indicate that Blimp1 favors cell invasion by altering focal adhesion dynamics and that FAK activation and expression regulation may depend on Blimp1 expression levels.

### Blimp1 expression is required for tumor growth and lung metastases formation *in vivo*

**Figure 4:**
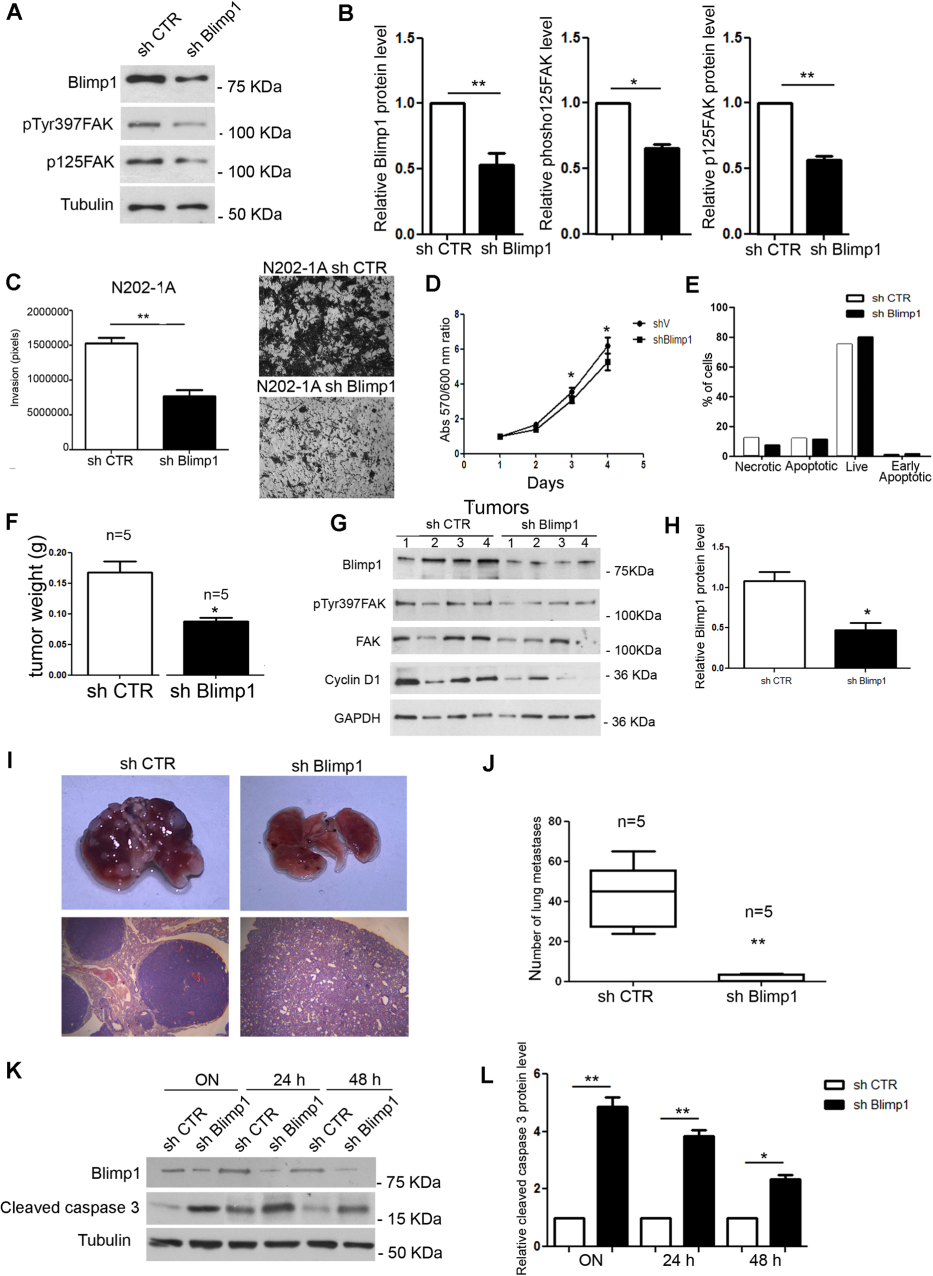
Blimp1 silencing impairs cell motility and *in vivo* metastasization of N202-1A cells. (**A**) N202-1A cells were transduced with a mouse shRNA sequence for Blimp1 and total cell extracts were probed for Blimp1, phospho-FAK(Tyr397) and p125FAK in a western blot analysis. Tubulin was used as the loading control. (**B**) Densitometric analyses of Blimp1, phospho-FAK(Tyr397) and FAK protein levels in at least three independent experiments are shown (mean ± s.e.m). Protein modulation was calculated relative to control cells and normalized on tubulin as loading control. Statistical analyses were performed using the Student’s t-test (*p < 0.05, **p < 0.01). (**C**) N202-1A control and sh Blimp1 cells were subjected to transwell invasion assays for 72 hours both in the presence and the absence of 20% FBS. Cell invasion quantification was performed over three different experiments. Results are presented as mean ± s.e.m. of the area covered by invaded cells (left panel). Representative images of invaded cells after 72 hours, fixed and stained with Crystal violet are reported (**p < 0.01) (right panel). Magnification 5X. (**D**) Cell viability of N202-1A control and sh Blimp1 cells in 2D adherent conditions (*p < 0.02). (**E**) Cell survival of N202-1A control and sh Blimp1 cells in 2D adherent conditions. (**F**) Weight quantification of tumors derived from NSG mice injected into the mammary fat pad with control or sh Blimp1 N202-1A cells (**p < 0.05). (**G**) Total cell extracts of tumors from control and sh Blimp1 N202-1A cells were probed for Blimp1, phosphoFAK(Tyr397), p125FAK, cyclinD1 in a western blot analysis. GAPDH was used as loading control. (**H**) Densitometric analyses of Blimp1 protein level modulation was calculated relative to control and normalized to GAPDH is shown (mean ± s.e.m) (**p < 0.01). (**I**) Pictures of representative whole lung and H&E staining of lung metastasis from NSG mice 5 weeks after the tail vein injection of either the N202-1A control or sh Blimp1 cells are shown. (**J**) Total number of metastases per lung is shown as box and whisker plots with median and minimum/maximum (n = 5 mice per group). Statistical analyses were performed using the Student’s t-test (**p < 0.01). (**K**) Total cell extracts from control and sh Blimp1 N202-1A cells grown in a suspension overnight, for 24 or 48 hours were probed for Blimp1 in a western blot analysis. Tubulin was used as the loading control. (**L**) Densitometric analysis of cleaved caspase 3 levels in at least three independent experiments is shown (mean ± s.e.m). Cleaved caspase 3 protein modulation was calculated relative to control cell level and normalized to tubulin as the loading control. Statistical analyses were performed using the Student’s t-test (*p < 0.05, **p < 0.01).

No data on the role of Blimp1 in *in vivo* solid tumors are currently available. As MCF10A.B2 cells do not support *in vivo* growth, we chose N202-1A cells as experimental model to investigate this aspect. N202-1A cells derive from HER2/neu transgenic mice, express high levels of p130Cas and require p130Cas expression for *in vivo* tumor formation and dissemination^5^. To examine the relevance of Blimp1 in *in vivo* tumorigenesis, N202-1A cells were infected with control or lentiviral vectors carrying Blimp1 shRNA. As shown in Fig. 4A, an efficient knock-down of Blimp1 was observed along with a significant reduction of FAK protein activation and expression levels (Fig. 4A and B). Moreover, silencing of Blimp1 in N202-1A cells was sufficient to reduce cell invasion *in vitro* (Fig. 4C), further confirming that Blimp1 is critical for p130Cas/ErbB2-driven invasion in another cell model of breast cancer. In addition, silencing of Blimp1 is sufficient to reduce significantly N202-1A cell viability in adherent conditions (Fig. 4D) but does not alter cell survival (Fig. 4E), indicating that cells silenced for Blimp1 proliferate less than control cells.

To investigate the possible contribution of Blimp1 to mammary tumor growth, Blimp1-silenced and relative control N202-1A cells were orthotopically injected into the mammary fat pad of NSG immune-compromised mice. After three weeks, tumors were excised and measured. Notably, in Blimp-1-depleted tumors the weight was significantly reduced compared to controls (Fig. 4F). Western blot analysis of tumor protein extracts revealed that Blimp1 was effectively knocked down *in vivo*, and its down-regulation correlated with decreased FAK activation and expression and decreased expression of Cyclin D1 (Fig. 4G,H). Thus, these experiments demonstrate that Blimp1 expression impairs ErbB2/p130Cas-dependent tumor growth *in vivo*, by affecting cell proliferation.

Next, to explore the involvement of Blimp1 in tumor progression, experimental metastasis formation assays were performed by injecting N202-1A control and Blimp1 silenced cells into the tail veins of NSG mice. The mice were sacrificed and their lungs recovered to assess metastasis formation after 5 weeks. As displayed in Fig. 4I and J, stronger lung nodule reduction was detected in lungs derived from shBlimp1 cell injected mice than in controls. In a further experiment, N202-1A control and Blimp1 silenced cells were seeded on low adhesion plates and apoptosis was evaluated in order to assess whether the impaired lung colonization of Blimp1 silenced cells was due to increased cell death. Indeed, cleaved Caspase-3 expression levels increased only in Blimp1 silenced cells, indicating that these cells are more susceptible to apoptosis induced by cell detachment and thereby less efficient in driving lung colonization (Fig. 4K,L). These data indicate that Blimp1 supports N202-1A proliferation under adherent conditions and resistance to anoikis. It is conceivable that these two mechanisms may co-exist during tumorigenesis, leading to increased tumor growth and ability to give rise to metastasis.

### miR-23b as a new regulator of Blimp1 in breast cancer invasion

Once it has been demonstrated that Blimp1 overexpression is crucial for driving invasion in breast epithelial cells, the next step is looking for more selective mechanisms that can block its expression. It was therefore decided that we evaluate the possible interactions of miR-23b in this context, as it has previously been demonstrated to be downmodulated in invasive acini^4^, and that Blimp1 was predicted to be one of its putative targets (TargetScan v. 6.2).

**Figure 5:**
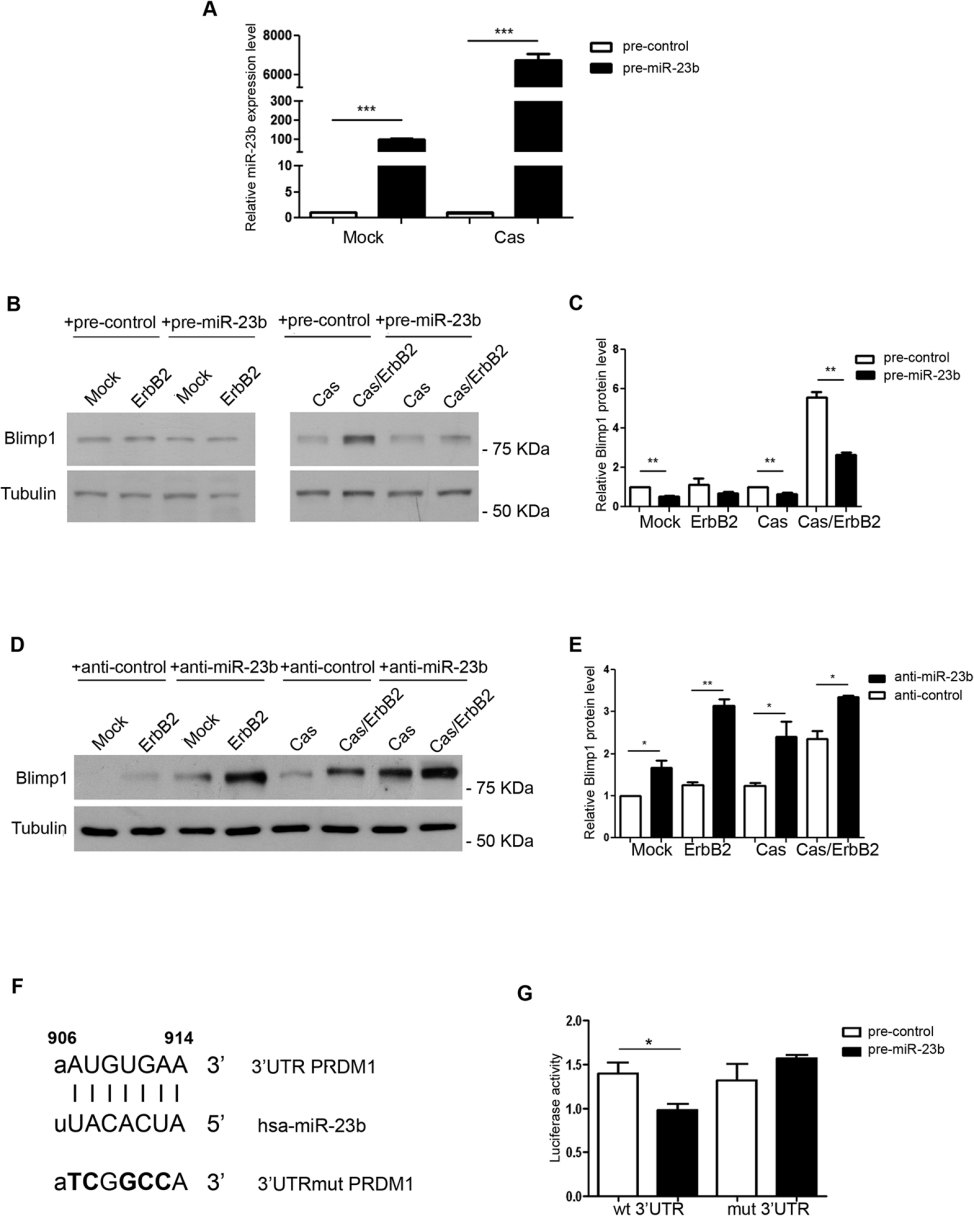
miR-23b directly binds to Blimp1 and regulates its expression. (**A**) miR-23b levels were evaluated using qRT-PCR in MCF10A.B2 Mock and Cas cells 48 hours after transfection with either the miR-23b precursor (pre-miR-23b) or negative controls (pre-control). Results were calculated as fold changes (mean ± s.e.m.) relative to controls, normalized to U44 (***p < 0.001). (**B**) Total cell extracts from Mock and Cas cells, that had been treated with 1 µM AP1510 or left untreated, 48 hours after transfection with either the miR-23b precursor (pre-miR-23b) or negative controls (pre-control) were probed for Blimp1 in a western blot analysis. Tubulin was used as the loading control. Blimp1 protein modulation was calculated relative to Mock level and normalized to tubulin as the loading control. (**C**) Densitometric analysis of protein levels in at least three independent experiments is shown (mean ± s.e.m.). Statistical analysis was performed using the Student’s t-test (**p < 0.01). (**D**) Total cell extracts from Mock and Cas cells, that had either been treated with 1 µM AP1510 or left untreated, 24 hours after transfection with either the miR-23b inhibitor (anti-miR-23b) or negative controls (anti-control) were probed for Blimp1 in a western blot analysis. Tubulin was used as the loading control. Blimp1 protein modulation was calculated relative to Mock level and normalized to tubulin as the loading control. (**E**) Densitometric analyses of protein levels in at least three independent experiments are shown (mean ± s.e.m.). Statistical analyses were performed using the Student’s t-test (*p < 0.05, **p < 0.01). (**F**) Seed matches and mutated binding sites (3′UTRmut PRDM1) of miR-23b in the 3′UTR of PRDM. Black bars indicate the seed matches, while bold letters indicate mutation sites. (**G**) Luciferase assays in HEK293 cells cotransfected with either wild-type or mutant PRDM1 3′UTR reporter constructs, together with miR-23b precursors (pre-miR-23b) and negative controls (pre-control).

MCF10A.B2 control and p130Cas overexpressing cells were either transiently transfected with a miR-23b precursor (pre-miR-23b) or negative controls (pre-control) and then either treated with AP1510 to drive the activation of ErbB2 or left untreated. miR-23b and Blimp1 expression were assessed 48 hours post transfection (Fig. 5A–C). The results shown in Fig. 5B and C indicate that miR-23b overexpression leads to a 50% reduction in Blimp1 expression in invasive Cas/ErbB2 conditions. Furthermore, the transfection of a specific miR-23b inhibitor (anti-miR-23b) increases Blimp1 expression both in Mock and Cas cells whether treated with the ErbB2 homodimerizer or not (Fig. 5D,E). This suggests that miR-23b acts as a regulator of Blimp1 in normal, transformed and invasive breast epithelial cells.

In order to determine whether this regulation is a consequence of the direct binding of miR-23b on PRDM1 3′UTR, we used a reporter vector containing part of the 3′UTR of PRDM1, from nucleotide 538 to 2419^31^, to perform luciferase reporter assays in HEK293T cells transfected with either pre-miR-23b or pre-control sequences. As shown in Fig. 5F, luciferase expression, which is driven by the 3′UTR of Blimp1, significantly decreased upon pre-miR-23b overexpression. Point mutations were inserted in the first miR-23b seed from nucleotide 907 to 914, as indicated in Fig. 5G, and luciferase expression was evaluated in the presence of miR-23b overexpression (Fig. 5F) as a means of evaluating miR-23b direct binding on Blimp1 3′UTR sequences. Taken together, these data indicate that miR-23b directly binds and regulates Blimp1 expression.

### miR-23b impairs p130Cas/ErbB2 invasion by negatively regulating Blimp1 expression

**Figure 6:**
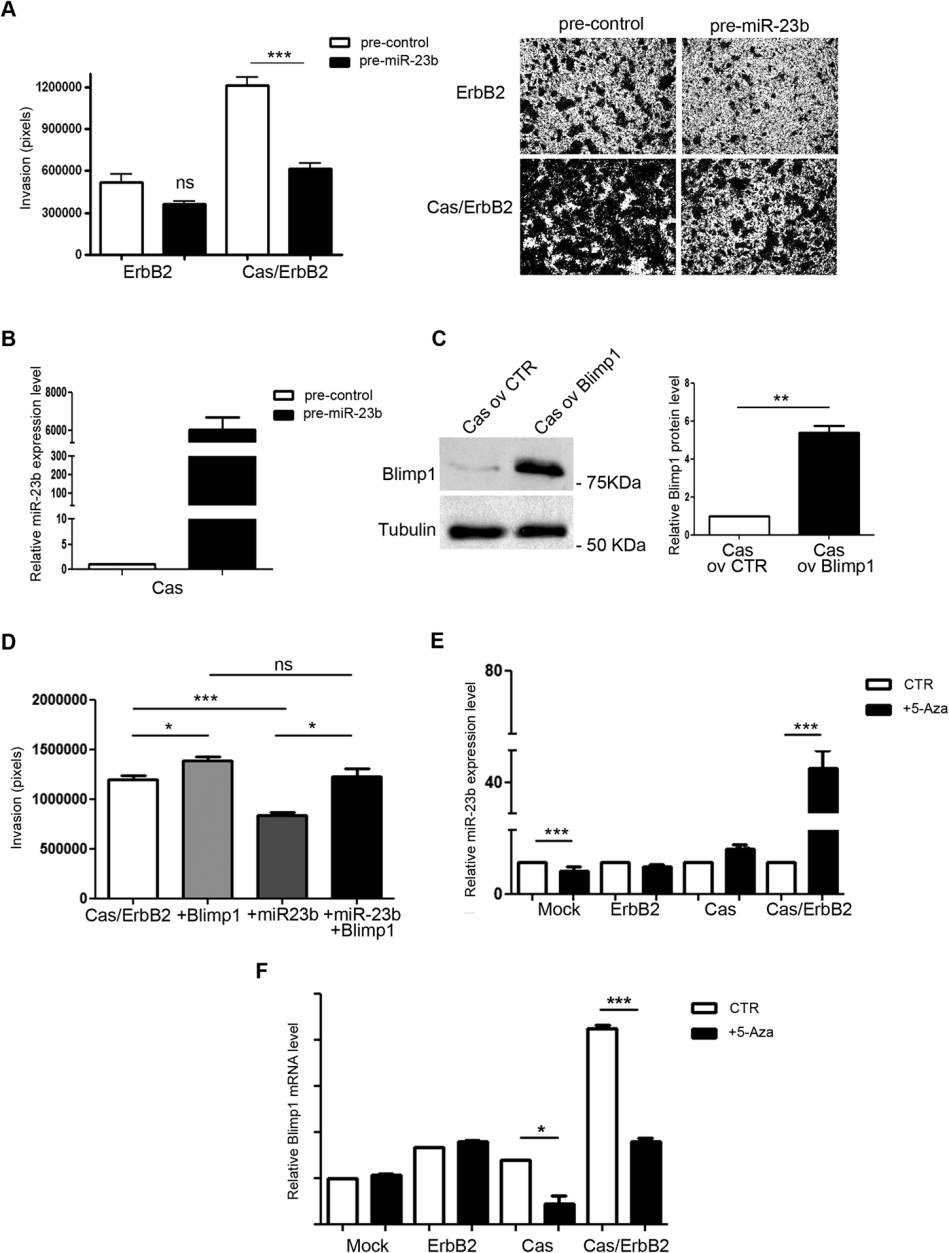
Blimp1 is a new negative target of miR-23b that impairs cell invasion. (**A**) MCF10A.B2 Mock and Cas cells 24 hours after transfection with either the miR-23b precursor (pre-miR-23b) or negative controls (pre-control) were subjected to transwell invasion assays for 48 hours in the presence of 1 µM AP1510. Cell invasion quantification was performed across three different experiments. Results are presented as mean ± s.e.m. of the area covered by invaded cells. Representative images of invaded cells after 48 hours, fixed and stained with Crystal violet are reported (***p < 0.001). Magnification 5X. (**B**) qRT-PCR of miR-23b in MCF10A.B2 Cas cells showing the levels of miR-23b expression 48 hours after pre-miR-23b transfection (pre-miR-23b) as compared to control (pre-control) transfected cells. Results were calculated as fold changes (mean ± s.e.m.) relative to controls, normalized to U44 (**C**) MCF10A.B2 Cas cells overexpressing Blimp1 protein lacking its 3′UTR were probed for Blimp1 in a western blot analysis. Tubulin was used as the loading control. Densitometric analyses of protein levels in at least three independent experiments are shown (mean ± s.e.m.). Statistical analyses were performed using the Student’s t-test (**p < 0.01). (**D**) MCF10A.B2 Cas/ErbB2 cells were subjected to transwell invasion assays for 48 hours. Cas/ErbB2 cells were either transduced to overexpress Blimp1 protein (+Blimp1), transfected with the precursor miR-23b for 24 hours (+miR-23b) or transduced to overexpress Blimp1 protein and transfected with the precursor of miR-23b contemporary (+Blimp1, +miR-23b). Statistical analyses were performed at least on three independent experiments using the Student’s t-test (*p < 0.05, ***p < 0.001). (**E**) qRT-PCR of miR-23b after treatment of MCF10A.B2 Mock, ErbB2, Cas, Cas/ErbB2 cells with 10 µM 5-Aza or DMSO for 48 hours. Results were calculated as fold changes (mean ± s.e.m.) relative to controls, normalized to U44 (***p < 0.001). (**F**) qRT-PCR of Blimp1 after treatment of MCF10A.B2 Mock, ErbB2, Cas, Cas/ErbB2 cells with 10 µM 5-Aza or DMSO for 48 hours. Results were calculated as fold changes (mean ± s.e.m.) relative to controls, normalized to 18S (*p < 0.05, ***p < 0.001).

The role of miR-23b in breast cancer invasion is still the subject of some controversy since both tumor suppressor and oncogenic activities have been reported as occurring in invasive breast cancer cells^32, 33^. It has been described that miR-23b is downmodulated in invasive acini, leading us to speculate that miR-23b may play a tumor suppressor role in the p130Cas/ErbB2 breast cancer model. Interestingly, the ectopic expression of miR-23b in Cas/ErbB2 MCF10A.B2 cells led to significantly lower migratory and invasive capacity than shown in the controls (Fig. 6A).

To ascertain whether the anti-migratory effect exerted by miR-23b was due to Blimp1 expression downmodulation, pre-miR-23b was transiently transfected in Cas overexpressing cells (Fig. 6B) that had previously been transduced with lentiviral vectors for the overexpression of Blimp1 lacking its 3′UTR or with empty vectors (Fig. 6C). Invasion was assessed by performing Transwell invasion assays (Fig. 6D). Interestingly, Blimp1 overexpression in Cas/ErbB2 cells leads to increased migratory and invasive capacities, while miR-23b overexpression-driven decreased cell invasion is rescued upon concomitant Blimp1 overexpression. These data suggest that miR-23b is an important regulator of Cas/ErbB2 breast cancer invasion and that it does so via the regulation of Blimp1 expression levels.

Moreover, the possible mechanisms that cause miR-23b downmodulation in p130Cas/ErbB2 invasive cells were evaluated. Recent evidence indicates that the miR-23b regulatory region in the genome contains a number of CpG islands that are distributed at a high density upstream of the transcription start site which could be affected by methylation and lead to the silencing of transcription^34^. Therefore, we tested the effects of a compound that inhibits CpG islands methylation (5′-Aza) on miR-23b expression in Mock and Cas cells, activated or not for ErbB2. Importantly, inhibition of DNA methylation resulted in the up-regulation of miR-23b expression together with a reduction of its target Blimp1 mRNA in p130Cas/ErbB2 cells (Fig. 6E and F). Taken together, these data suggest that epigenetic regulation might be a possible mechanism of miR-23b downmodulation in p130Cas/ErbB2 cancer cells.

### Blimp1/PRDM1 expression levels correlate with the presence of multiple metastases

The *in vitro* and *in vivo* data presented above point out Blimp1 as a mediator of breast cancer progression. Therefore, Blimp1 expression levels in human breast tumors were evaluated as a means of further understanding their involvement in metastases formation.

**Figure 7:**
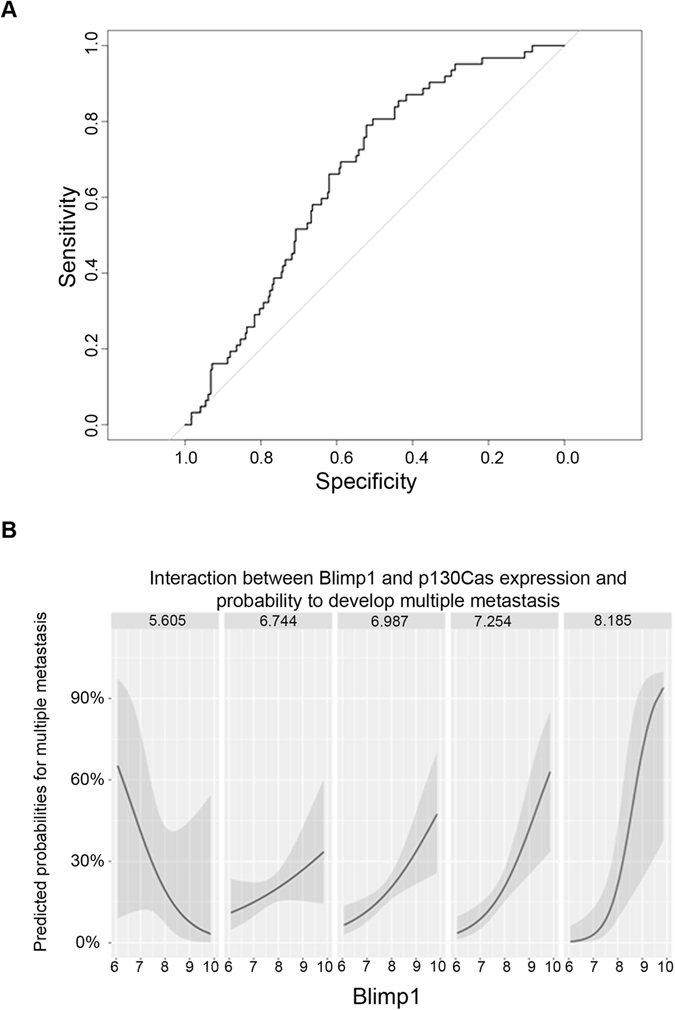
The interaction between Blimp1 and p130Cas levels positively correlates with the risk of developing multiple metastases. (**A**) ROC curve representing the prediction results of the logistic regression that discriminates between patients with single or multiple metastases using PRDM1 and BCAR1 expression levels and their interaction as predictors. (**B**) Effects plots of the regression. A separate plot of the fit is depicted for different levels of BCAR1, from left to right respectively: minimum, 1^st^ quartile, median, 3^rd^ quartile and maximum. In presence of higher expression of BCAR1, the effect of PRDM1 levels on the predicted probabilities becomes positive and progressively larger.

To investigate the relationship between Blimp1 and p130Cas expression levels and their effect on the metastatic process, we fitted a logistic model to discriminate between patients with multiple distant or lymph nodal metastases (n = 295) and those with a single metastasis (n = 62), using Blimp1 and p130Cas expression levels and their interaction as predictors^35^. We obtained an AUC of 0.66 (Fig. 7A) but more importantly a significant p-value for the interaction term, which indicates a higher risk of developing multiple metastases for patients with high expression of p130Cas and Blimp1 at the same time (Fig. 7B).

Blimp1 expression levels also change with different grading (Supplementary Figure 9) and staging of tumors (data not shown) (Kruskal-Wallis test p-values < 0.05). On the whole cohort we were not able to detect a significant effect on survival, however on the subset of patients with at least a metastasis, PRDM1 and BCAR expression levels are predictive of the overall survival, with worse prognoses for patients with higher expression (data not shown, PRDM p-value 0.02, 95% IC of HR 1.037–1.499, multivariate Cox regression). These results indicate that Blimp1 expression positively correlates with a higher probability of developing multiple metastasis in patients with high p130Cas, supporting the major role played by p130Cas in Blimp1-mediated invasion.

## Discussion

We herein describe the role of the transcriptional repressor Blimp1 in p130Cas/ErbB2 breast cancer invasion in *in vitro* and *in vivo* models for the first time. In particular, we demonstrate that Blimp1 is highly expressed during p130Cas/ErbB2 dependent invasion in MCF10A.B2 cells and that the modulation of its expression is sufficient to severely impair tumor invasiveness *in vitro* and lung metastasis formation *in vivo*.

Specifically, a 3D model of MCF10A.B2 cells was used to demonstrate that high levels of Blimp1 expression specifically occur in invasive acini in which p130Cas is overexpressed and ErbB2 is activated.

Although the ability of the transcriptional repressor Blimp1 to influence cell migration has already been explored in the immune system, where its conditional knock-out in CD8+ T cells impairs the mobilization of virus-specific CD8+ T cells to the site of infection^36^, the amount of data available on the role of Blimp1 in non-hematopoietic cells is still limited. Indeed, it has been shown that the downmodulation of Blimp1 in solid tumors leads to impaired cell migration both in non-small cell lung cancer and in ERα positive breast cancer cell lines, where Blimp1 represses the BMP5 protein^16, 18^.

This paper also shows proof that MAPK inhibition results in Blimp1 expression downmodulation and in the reduction of both multiacinar structures and invasive protrusions in p130Cas/ErbB2 cells. The involvement of Erk1/2 MAPK activation signaling in p130Cas/ErbB2 invasion in the 3D cell model had already been described by our group^8^, however, this investigation provides further insight, as Blimp1 is described as a downstream effector of Erk1/2 activation.

The mechanisms via which MAPKs regulate the expression of Blimp1 have also been described; Erk1/2 activation in B cells inhibits the PAX5 transcription factor which acts as a negative regulator of Blimp1 expression, thus leading to its enhanced expression^37^. However, preliminary data suggest that Pax5 is not involved in MAPK-dependent Blimp1 expression in our work, indicating that additional mechanisms, which will require further investigation, may well account for the modulation of Blimp1 expression in solid tumors.

Our data also indicate that Blimp1 overexpression is sufficient to provide p130Cas overexpressing MCF10.B2 mammary cells with invasive features, whereas its overexpression in ErbB2 only activated cells does not promote invasion. These data indicate that Blimp1 requires p130Cas expression to trigger invasion, whereas the activation of ErbB2 is dispensable for invasion in the absence of p130Cas. It is worth noting that *in silico* analyses of breast cancer patients support these data by pointing out that high levels of p130Cas and Blimp1 correlate with multiple metastasis. Our data also indicate that Blimp1 expression modulation results in an alteration in FAK activation and expression. The well-established role of FAK and p130Cas in cell migration and invasion^23, 38, 39^, coupled with the fact that our findings identify FAK as an effector of Blimp1-dependent invasion lead to our speculation that Blimp1 overexpression is sufficient to induce invasion in p130Cas overexpressing mammary cells by leading to high FAK activation and expression, the consequent activation of p130Cas-dependent pathways, which, in turn, result in focal adhesion reorganization. However, Blimp1 overexpression in ErbB2 without the presence of high p130Cas levels does not provide a proper substrate for FAK-induced invasion. Intriguingly, *in silico* analyses that searched for Blimp1 binding sites on FAK promoters showed that PRDM1/Blimp1 binding sites are present in the proximity of FAK promoters, which further supports the relevance of Blimp1/FAK/p130Cas in cell invasion. These observations suggest that Blimp1 can act as a transcription factor that regulates FAK expression and shed additional light onto FAK transcriptional regulation.

On the other hand, our data also show that Blimp1 expression can be directly modulated by mir-23b. In particular, we herein describe, for the first time, that miR-23b is a direct Blimp1 negative modulator in solid tumors. In this context, we also demonstrate that miR-23b is a tumor suppressor miRNA in p130Cas/ErbB2 cells and that Blimp1 expression downmodulation is critical for miR-23b-dependent cell invasion impairment. Several reports have highlighted that cancer cell invasion and migration is also regulated by microRNAs (miRNAs); an important class of signaling modulators that are often deregulated in human cancers^40^. miR-23b is a highly conserved miRNA that belongs to the miR-23b-27b-24-1 cluster (9q22.32), which is found intronically in the aminopetidase O gene^19^. miR-23b is generally described as mediating tumor suppression in human cancers, but it also affects tumor formation and progression, according to tissue and cell contexts. In particular, miR-23b is found to be downmodulated in a number of human cancers including breast, bladder, prostate and pancreatic cancers^20^. It has been described that miR-23b directly regulates target genes involved in cell migration, such as Zeb1, Src and PAK2^20, 33, 40^. Moreover, miR-23b has recently been reported to target Blimp1 in B cells following histone deacetylases inhibition in antibody and autoantibody responses^41^.

**Figure 8:**
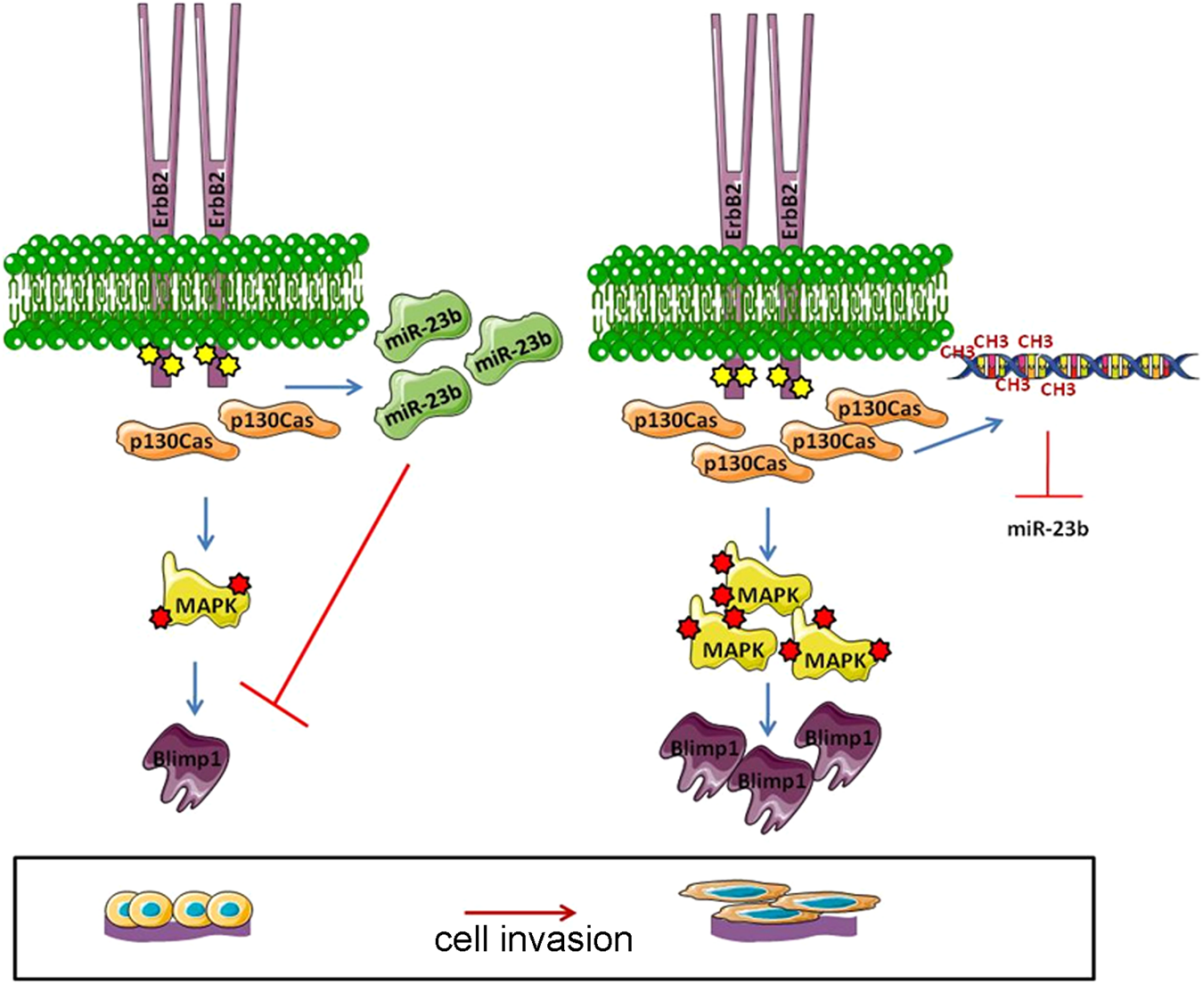
Graphical summary of the molecular mechanism leading to p130Cas/ErbB2/Blimp1 cell invasion. (**A**) In MCF10A.B2, the activation of ErbB2, in the absence of p130Cas overexpression, is not sufficient to increase Erk1/2 MAPKs activation and lower miR-23b expression to a threshold level that allows Blimp1 expression to occur (*left panel*). By contrast, ErbB2 activation and concomitant p130Cas overexpression strongly enhance Erk1/2 MAPKs activation and miR-23b promoter methylation, resulting in increased Blimp1 expression and, in turn, cell invasion (*right panel*).

Interestingly, we also demonstrate that miR-23b downmodulation is regulated by the methylation process in p130Cas/ErbB2 cells, which is in line with recently reported results in cervical cancer where several CpG islands were found to be part of the miR-23b regulatory region^34^. This work describes, for the first time, that p130Cas and ErbB2 are able to influence epigenetic programs, such as methylation. On the basis of our data, we can speculate that increased DNA methylases expression or activation might occur in p130Cas/ErbB2 cells leading to mir-23b methylation. However, further research will be required to identify the specific mechanism through which miR-23b is regulated by methylation and the specific chromatin remodeling modifiers that are selectively induced upon p130Cas/ErbB2 cooperation. In conclusion, Blimp1 has been identified as a crucial regulator of p130Cas/ErbB2-mediated invasiveness *in vitro* and *in vivo* and has thus provided new insight into Blimp1 expression regulation and its mechanism of action. We have described two mechanisms by which p130Cas and ErbB2 synergism is able to induce Blimp1 expression; MAPK pathway activation and miR-23b downmodulation (See Fig. 8). Moreover, we have demonstrated, for the first time, that the Blimp1 protein is a prometastatic target of the tumor suppressor miR-23b in invasive breast cancer. Therefore this study can pave the way for the development of a miRNA-based therapeutic tool that can be used to treat p130Cas/ErbB2 aggressive tumors.

## Methods

### Cell lines and Cell culture

MCF10A.B2 cells were kindly provided by Dr Muthuswamy^6^ and maintained as described in ref. 4, MCF10A.B2 Mock and MCF10A.B2 Cas were generated as described in Cabodi *et al*., 2011^5^. MCF10A.B2 without activation of ErbB2 was referred in the text as Mock, MCF10A.B2 with activation of ErbB2 as ErbB2, MCF10A.B2 overexpressing p130Cas without activation of ErbB2 as Cas, MCF10A.B2 overexpressing p130Cas with activation of ErbB2 as Cas/ErbB2. HEK293T were cultured in DMEM-10% FBS (Thermo Fisher Scientific), and N202-1A cells in DMEM-20% FBS (Thermo Fisher Scientific). All used cell lines were authenticated in the last 6 months by BMR Genomics (Padova, Italy), using the CELL ID System (Promega, Madison, WI).

MCF10.B2 and N202-1A cell populations were used for all the experiments described.

### Three-Dimensional Culture

Three-dimensional morphogenic assays were performed according to instructions found below; http://muthuswamylab.cshl.edu/ml_protocols.html.

For the inhibition of PI3K/Akt, Erk1/2 MAPK pathway acini were treated with 10 µM LY294002 (Calbiochem) and 25 µM PD98059 (Sigma) either with or without 1 µM AP1510 homodimerizer (A/A Homodimerizer, Clontech) at day 10. No off-target effects were reported after treating MCF10.B2 cells with 25–50 μM PD98059^8, 42–44^.

### Transient Transfections of pre-miRs and anti-miRs

For transient transfection experiments, 0.8 × 10^6^ MCF10A.B2 Mock and MCF10A.B2 Cas cells were seeded in a 6 well plate and the day after, transfected with either 75 nM pre-miR or 100 nM anti-miR using the Lipofectamine 2000™ reagent (Thermo Fisher Scientific Scientific), according to manufacturer’s instructions. 1 μM AP1510, or ethanol for the control, was added to the medium. Cells were tested for overexpression/downmodulation 24 and 48 hrs later.

### Luciferase assays

Cells (1.5 × 10^5^) were cotransfected with 25 ng of the pSIC.PRDM1.3′UTR.538-2419 reporter construct which contained part of the 3′UTR of PRDM1 from nucleotide 538 to 2419^31^, and either 75 nM of pre-miR-23b-3p or 100 nM anti-miR using Lipofectamine2000 (Thermo Fisher Scientific). Lysates were collected 24–48 h after transfection and Firefly and Renilla luciferase activities were measured using a Dual Luciferase Reporter System (Promega). Where indicated, Blimp1 3′UTR was mutagenized at the first miR-23b recognition site (nucleotides 907–914) using the QuikChange Lightning Site-Directed Mutagenesis Kit (Stratagene), according to manufacturer’s instructions.

### Antibodies and reagents

Erk1/2 (T202/Y204), Akt (S473), Fak (397Tyr) phosphor-antibodies and Erk1/2, Akt, Blimp-1/PRDI-BF1 and cleaved caspase-3 antibodies were from Cell Signaling. Vinculin antibody was produced in our laboratory. Fak polyclonal antibodies were from Abcam. GAPDH antibodies from Millipore, p130Cas antibodies were from BD Biosciences. Matrigel and collagen bovine I were from Corning. Peroxidase-conjugated secondary antibodies were from GE Healthcare. Pre-miR™ microRNA Precursor Molecules for Negative Control #1, Hsa-miR-23b (PM10711), anti-miR™ miRNA Inhibitor Negative Control #1, anti-miR™ miRNA Inhibitor Hsa-miR-23b-3p (AM10711) (Ambion) and TaqMan® MicroRNA Assays for Hsa-miR-23b (ID 000399), U44 snRNA (ID 001094) were from Applied Biosystems.

### Treatment of cell lines with 5′-Aza-2-deoxycytidine (5-Aza)

MCF10A.B2 Mock and Cas cells were seeded in 6-well plates (4 × 10^5^ cells/well). After 24 h, 10 μM 5-Aza (Sigma) dissolved in DMSO were added to fresh culture medium. 1 µM AP1510 was added an hour and a half later, where required. The cultures were incubated at 37 °C in 5% CO2 for 48 h. Control cells were treated with DMSO.

### Cell viability and survival

Cell Titer Blue assay was performed following manufacturer’s recommendations. Briefly, Cell Titer Blue was mixed to the culture medium at a concentration of 20% (v/v). At each time point, N202-1A cells cultured in 48-well plates were washed with PBS to remove the medium, then 500 μl of the Cell Titer Blue/cell culture medium mixture was added to each sample. After 1 hour, 100 μl were removed from the plates and the absorbance was measured in a 96-well plate using GloMax-Multidetection System (Promega, Italy). Cell survival was evaluated by annexin V staining followed by FACS analysis.

### Protein and RNA extraction, Immunoblotting and qRT-PCRs

Proteins, RNA extraction, immunoblotting were performed as previously described in ref. 4. qRT-PCRs for miR-23b detection were performed using TaqMan® MicroRNA Assays (Applied Biosystems) on 10 ng total RNA, according to manufacturer’s instructions. For mRNA detection, 1 μg of DNAse-treated RNA (DNA-free™ kit, Ambion) was retrotranscribed using RETROscript™ reagents (Ambion) and qRT–PCRs performed using gene-specific primers, on a 7900HT Fast Real Time PCR System. Quantitative normalization was performed on the expression of U44snRNA. For PRDM1 qRT-PCRs, the isolated total RNAs were reverse transcribed using a High Capacity cDNA Reverse Transcription kit (Applied Biosystems). The cDNA was diluted 1:5 in RNAase free water. qRT-PCR was performed on cDNA using the 7900HT Fast Real-Time PCR System (Applied Biosystems) with TaqMan Universal PCR Master Mix (Applied Biosystems). Primers used:

PRDM1 RIGHT: ACGTGTGGGTACGACCTTG, PRDM1 LEFT: CTGCCAATCCCTGAAACCT, MUT3′UTR PRDM1RIGHT:TTAAAACATGGCATTTTTTTCTTCTGGCCGATTTCTGATAATACAATGGA, MUT3′UTRPRDM1LEFT:CGGCCACATGACTTTTGCATCCATTGTATTATCAGAAATCGGCCAGAAGAAAAAAATGCCATGTTTTAA).

Conditions were: 50 °C (2 min), 95 °C (2 min), followed by 45 cycles of 90 °C (15 s) and 60 °C (30 s). A total volume of 20 µl was used. Quantitative normalization was performed on the expression of endogenous control 18S (Thermo Fisher Scientific). The relative expression levels between samples were calculated using the comparative delta CT (threshold cycle number) method (2^−ΔΔCT^) using a control sample as reference point^45^.

### Immunofluorescence

Cells (2 × 10^5^) were seeded into a 24-well plate (BD Falcon) and were either treated with ethanol (vehicle) or 10 µM AP1510 the next day. After 48 hours, cells were rinsed with ice-cold PBS and fixed with 4% paraformaldehyde for 10 min at RT, followed by permeabilization with 0.25% Triton X-100. Cells were stained with the vinculin (1:1000) antibody for 1 hour at RT, then washed with PBS and incubated with Alexa 568-labeled anti-mouse secondary antibody (Red) (1:500) (Thermo Fisher Scientific Scientific) at RT for 30 minutes together with TRITC-conjugated Phalloidin (Green) (P2141, Sigma). After 4, 6-diamino-2-phenylindole (DAPI, Thermo Fisher Scientific Scientific) staining, slides were mounted using Pro-long Gold Antifade Reagen (Thermo Fisher Scientific Scientific). Images were captured on a HCX PL APO CS 63 × 1.5 OIL Leica TCS-SP5 II confocal microscope and analyzed using LASAF software (Leica).

### Lentiviral constructs

For PRDM1 overexpression, human PRDM1 cDNA (Source Bioscience) was cloned into the pLVX lentiviral vector and viral particle production was performed as previously described^5^. The PRDM1 shRNA library was purchased from GE Healthcare Dharmacon Inc. (TRC-Hs1.0 human clone ID: TRCN0000013612, TRC-Mm1.0 mouse clone ID: TRCN0000084714). For p130Cas expression, human p130Cas cDNA was cloned into the pCCL lentiviral vector and viral particle production was performed as described above.

### Transwell invasion assay

Transwell invasion assay were performed as described in ref. 4. Briefly, 24-transwell chambers (Corning Costar, Cambridge, MA) were coated with 50 microliters of 1:1 mix matrigel plus collagen. MCF10A.B2 Mock and Cas cells (10^5^) in 100 μl of serum-free medium were seeded into the upper chamber. DMEM-F12 (100 μl) with 5% horse serum plus AP1510 or ethanol for control, was added into the lower chamber as chemotactic stimulus. After 48 hrs, the migrated cells on the lower side of the membrane were fixed in 2.5% glutaraldehyde, stained with 0.1% crystal violet and photographed using an Olympus IX70 microscope. Invasion was evaluated by measuring the area occupied by migrated cells using the ImageJ software (http://rsbweb.nih.gov/ij/). For N202-1A cells, DMEM (500 μl) with 20% FBS, or with water for the control, was added to the lower chamber as a chemotactic stimulus and invasion was evaluated, as above, after 72 hours.

### *In vivo* assays

The use of animals was in compliance with the Guide for the Care and Use of Laboratory Animals published by the U.S. National Institutes of Health and approved by the Animal Care and Use Committee of the University of Turin and by the Italian Health Minister (authorization #1009/2016-PR). N202-1A control, Blimp1 silenced and Blimp1 overexpressing cells (10^5^) were injected intravenously into the tail veins of NOD scid gamma (NSG) mice. The lungs were harvested, paraffin embedded, sectioned and stained with hematoxylin and eosin after 5 weeks. Slides were analyzed and photographed using an Olympus IX70 microscope and metastases were counted on H&E sections. N202-1A control, Blimp1 silenced and Blimp1 overexpressing cells (10^5^) were also injected into the fat pad of NSG mice. After 3 weeks tumors were recovered and tumor weight was evaluated.

### Bioinformatics analysis

Binding sites for Blimp1 were searched using the PRDM1 ChiP-seq on HeLa-S3 cells of the UCSC Track “Transcription Factor ChIP-seq Uniform Peaks from ENCODE/Analysis” on the promoter (1500 bp upstream and 500 downstream of TSS) of FAK on the human genome (release hg19), then inside the resulting peak we identified a subsequence with a high log-likelihood for the Jaspar PWM MA0508.1. Log-likelihoods^46^ were computed using an ad-hoc C program and the background nucleotide frequencies were defined on human intergenic sequences.

For the *in silico* analyses of Blimp1 expression in breast cancer we used the METABRIC cohort of primary breast tumor samples, which comprises Illumina HT-12 expression data and several clinical parameters. Already normalized expression values in this cohort were downloaded from the European Genome-phenome Archive (EGAS00000000122: EGAD00010000434, original microarray data obtained in Curtis *et al*.^35^ was then re-processed and normalized by ref. 47), and a subset of 357 samples with at least a single metastasis (considering both distal and lymph nodes metastases) were further analyzed. All analyses were performed with R (A language and environment for statistical computing. R Foundation for Statistical Computing, Vienna, Austria. URL https://www.R-project.org), we used glm to fit the logistic model^48^, the pROC package to plot the corresponding ROC curve and its AUC^49^, sjPlot and effects packages to obtain the plots representing how BLIMP1 and BCAR1 expression levels interact with respect to the risk of developing multiple metastases (Lüdecke D. “sjPlot: Data Visualization for Statistics in Social Science”, (2017)).

### Statistical analysis

The results are representative of at least three independent experiments performed in triplicate and are expressed as means ± s.e.m. Statistical analysis of the data was performed using a Student’s t-test.

## Electronic supplementary material

supplementary Figure
