# Supplementary material for: Dysregulation of Blimp1 transcriptional repressor unleashes p130Cas/ErbB2 breast cancer invasion
Source: Sci Rep. 2017 Apr 25;7:1145. doi: 10.1038/s41598-017-01332-z (PMC5430666; doi:10.1038/s41598-017-01332-z)
Supplement: Supplementary file 1 — supplementary Figure [file 41598_2017_1332_MOESM1_ESM.pdf]

“Dysregulation of Blimp1 transcriptional repressor unleashes p130Cas/ErbB2 breast cancer invasion” (SREP-16-42496-T)

Marianna Sciortino[1], Maria del Pilar Camacho Leal[1], Francesca Orso[1], Elena Grassi[1], Andrea Costamagna[1], Paolo Provero[1], Wayne Tam[2], Emilia Turco[1], Paola Defilippi[1], Daniela Taverna[1], and Sara Cabodi[1*]
1: Department of Biotechnology and Health Sciences, University of Turin, Via Nizza 52, 10126 Turin, Italy
2: Department of Pathology and Laboratory Medicine, Weill Cornell Medicine, New York, NY, USA

Supplementary File

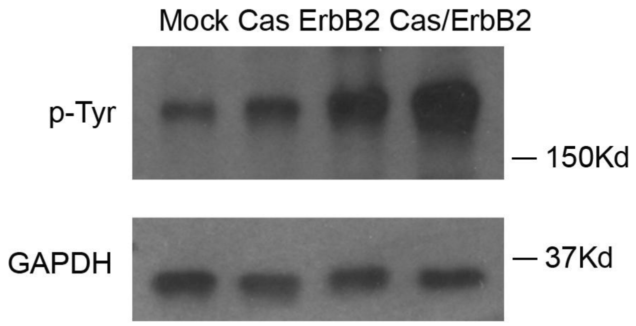

Supplementary Figure 1: ErbB2 activation in Mock and Cas acini, stimulated or not with AP1510.

MCF10.B2 wt (Mock) or overexpressing p130Cas (Cas) were treated with AP1510 (ErbB2 and Cas/ErbB2, respectively) and probe with phosphoTyrosine antibodies in a western blot analysis. GAPDH was used as the loading control.

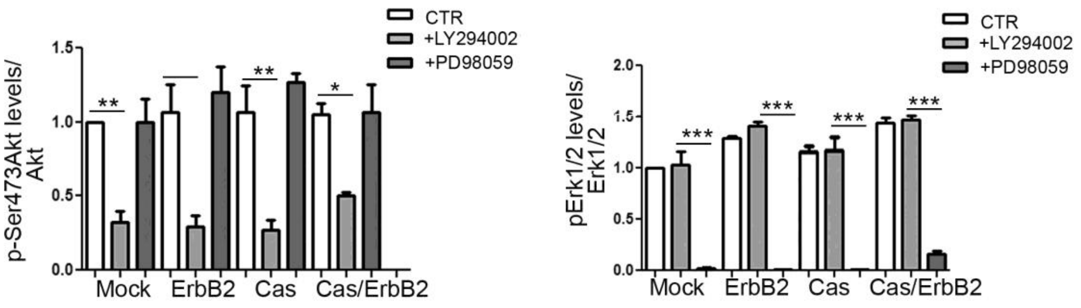

Supplementary Figure 2: Quantification of Akt and Erk1/2 inhibition upon treatment with LY294002 and PD98059. Densitometric analyses of activation of Akt (left panel) and Erk1/2 MAPKs (right panel) upon treatment with LY294002 and PD98059, respectively, of at least three independent experiments (mean±s.e.m) (p***<0.001, p**<0.01, p*<0.05). Akt and Erk1/2 modulation of activation was normalized to tubulin as the loading control.

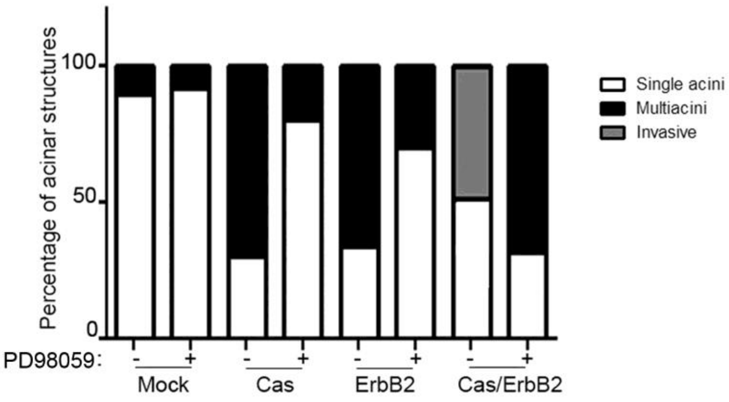

Supplementary Figure 3: Quantification of MCF10A.B2 structures in 3D upon treatment with Erk1/2 MAPKs inhibitor. Acinar structures (n= 100) were counted in Mock, Cas, ErbB2 and ErbB2/Cas cells in presence or absence of Erk1/2 MAPKs inhibitor (PD98059). As shown in the graph, Mock cells are mostly forming single acini in both treated and untreated conditions. The number of multiacinar structures in Cas overexpressing and ErbB2 activated cells is decreased by treatment with PD98059. The invasive structures that formed in Cas/ErbB2 cells are totally abrogated by treatment with PD98059.

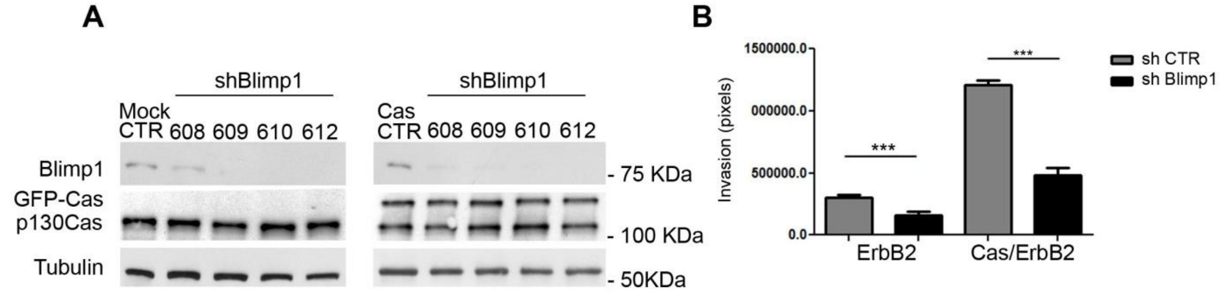

Supplementary Figure 4: Selection of Blimp1 sh sequences and evaluation of clone 608 in invasion assays.

(A) Mock and Cas cells were transduced using a number of Blimp1 sh RNAs and total cell extracts were probed for Blimp1 and p130Cas in a western blot analysis. Tubulin was used as the loading control **(B)** Mock and Cas cells, that had either been transduced with human sh Blimp1 clone 608 or scrambled control vector, were subjected to transwell invasion assays for 48 hours both in the presence and absence of 1 µM AP1510. Cell invasion quantification was performed across three different experiments. Results are presented as mean±s.e.m. of the area covered by invaded cells (***p<0.001).

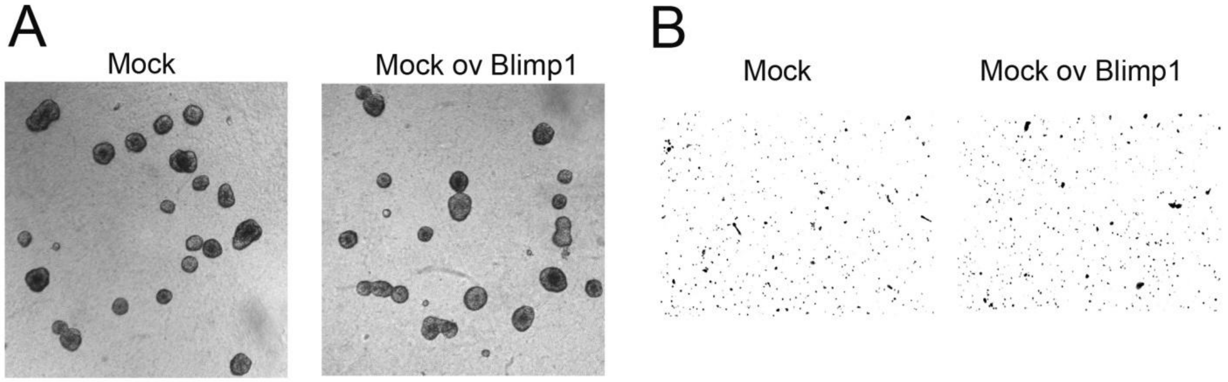

Supplementary Figure 5: Blimp1 overexpression in Mock cells does not induce multiacinar structures in 3D nor invasion in 2D.

(**a**) Representative phase contrast images of acinar structures taken at day 14. Magnification 20X. (**b**) Invasion of Mock and Blimp1 overexpressing Mock cells was evaluated by transwell invasion assays. Representative images of invaded cells after 48 hours, fixed and stained with Crystal violet are shown. Magnification 5X.

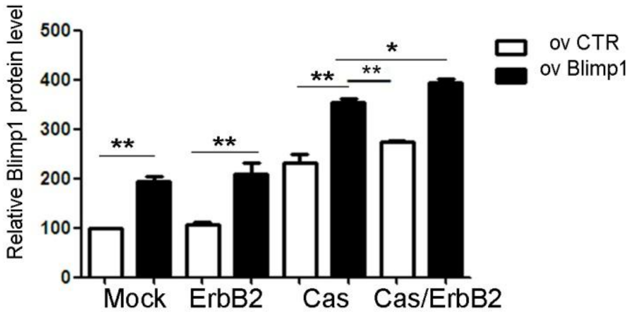

Supplementary Figure 6: Quantification of Blimp1 overexpression in Mock, ErbB2, Cas and Cas/ErbB2 MCF10A.B2 cells. Densitometric analyses of Blimp1 expression of at least three independent experiments (mean±s.e.m) ($p^{**}<0.01$, $p^{*}<0.05$). Blimp1 modulation of expression was normalized to GAPDH as the loading control.

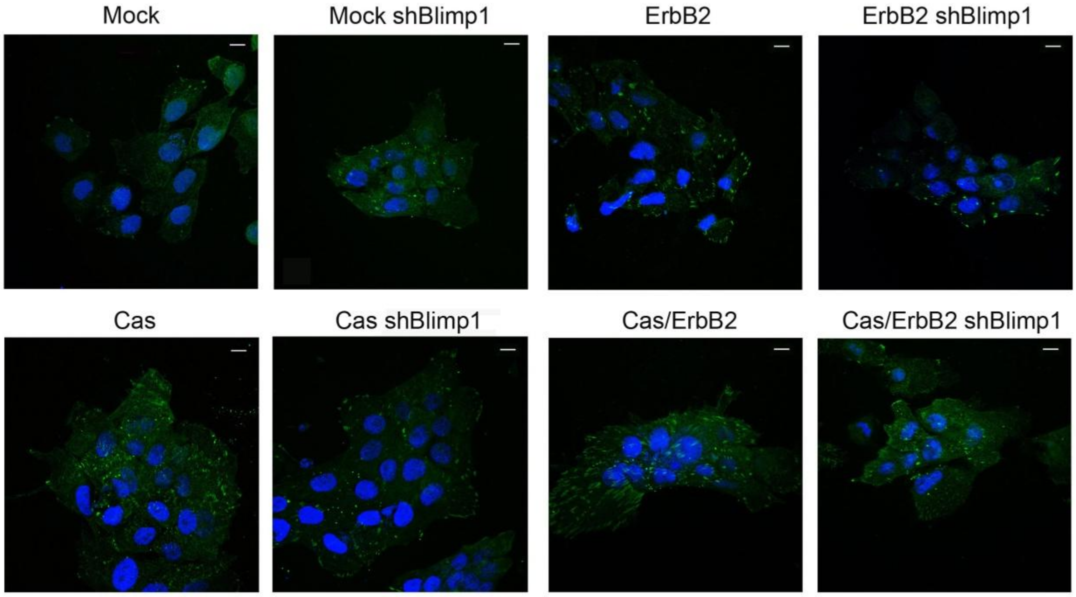

Supplementary Figure 7: FAK expression is down-modulated in Blimp1 silenced cells. Immunofluorescence staining for FAK (green) and DAPI (blu) of Mock and Cas MCF10A.B2 treated or not with 1µM AP1510 for 48 hours. Scale bars: 10 µm. Magnification 63X.

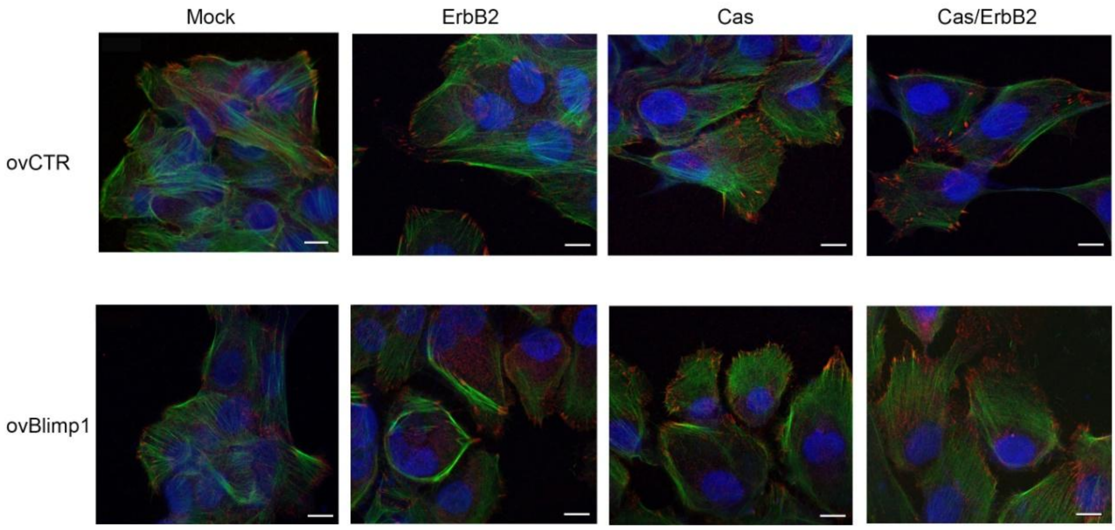

Supplementary Figure 8: Blimp1 overexpression enhances focal adhesions in Cas and Cas/ErbB2 invasive MCF10.B2 cells.

Immunofluorescence staining for vinculin (red) and phalloidin (green) of Mock and Cas MCF10A.B2 ctr or overexpressing Blimp1, treated or not with 1µM AP1510 for 48 hours. Scale bars: 10 µm. Magnification 63X.

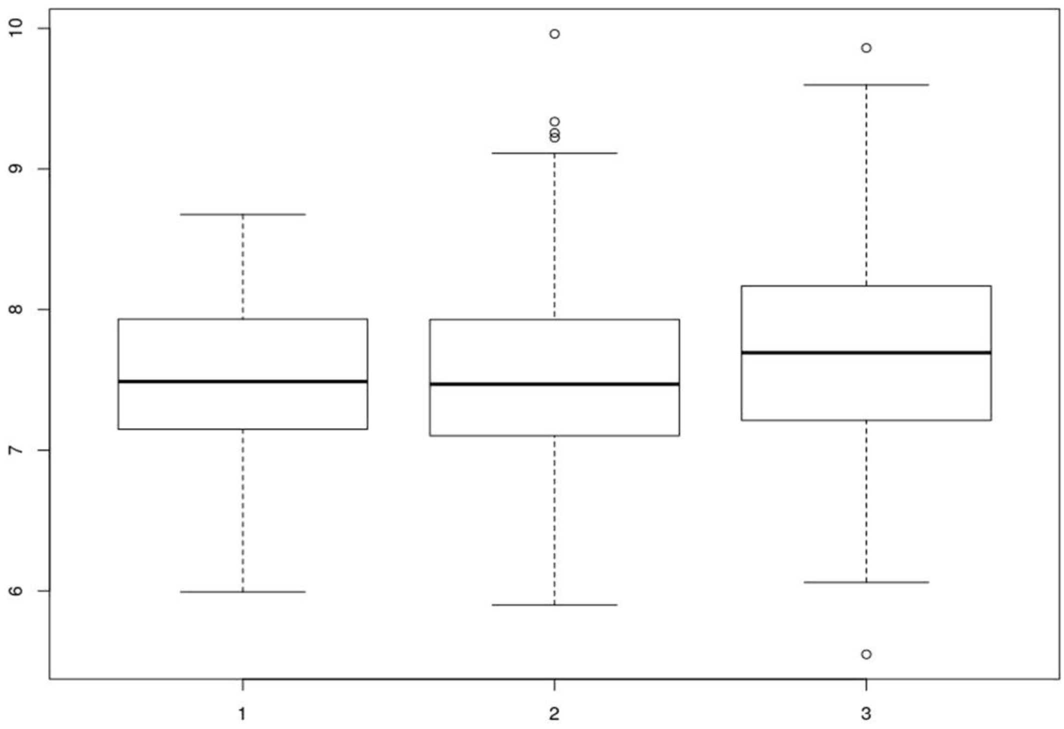

Supplementary Figure 9: Boxplot representing PRDM1 expression values across patients with different tumor grades.
